# Gastric microbiota transplantation enhanced the eradication of refractory *Helicobacter pylori* infection by modulating the gastric microbiota: a pilot study

**DOI:** 10.1128/spectrum.03263-24

**Published:** 2025-08-18

**Authors:** Jun Li, Lanfan Liang, Jian Ye, Yanping Miao, Kui Zhao, Yan Tian, Xiaohui Li, Xuemei Li, Xia Chen, Biao Wen, Ying He, Baijun Chen, Ling Qin, Yanwei Wang, Xiangsheng Fu

**Affiliations:** 1 Department of Gastroenterology, Clinical Medical College and the First Affiliated Hospital of Chengdu Medical College, Chengdu, China; The Chinese University of Hong Kong, Hong Kong, Hong Kong

**Keywords:** *Helicobacter pylori*, gastric microecology, gastric microbiota transplantation

## Abstract

Dysbiosis of the gastric microecology is implicated in various gastric diseases, with *Helicobacter pylori* (*H. pylori*) infection serving as a pivotal factor influencing the gastric microecological balance and vice versa. In this study, we investigated the novel effects of gastric microbiota transplantation (GMT) on gastric microecology and the potential of this treatment to enhance *H. pylori* eradication. We performed a metagenomic analysis of the microecological systems across different regions of the stomach, including the gastric fluid (GF), the gastric mucus layer (GML), and the gastric mucosa (GM). We initiated a clinical GMT intervention by transplanting microbial communities from healthy individuals' GML into patients exhibiting refractory *H. pylori* infection and chronic atrophic gastritis. Our findings demonstrated significant disparities in species richness among the GF, GML, and GM, with the GML exhibiting the highest diversity of unique microbial genera. *H. pylori* infection primarily influenced the relative species abundance within the GML community, without altering its fundamental composition. Clinically, GMT was well-tolerated by all recipients and showed substantial synergistic efficacy against refractory *H. pylori* infection, achieving a 100% eradication rate in all patients, and significantly alleviating symptoms in individuals with *H. pylori*-positive atrophic gastritis (*P* < 0.05). Compared with the gastric microbiota of *H. pylori*-negative patients, the gastric microbiota of *H. pylori*-positive patients treated with GMT exhibited closer alignment with those of healthy donors. In conclusion, GMT utilizing GML enhanced the eradication rate of refractory *H. pylori* infection and improved symptoms in patients with *H. pylori*-positive atrophic gastritis through modulating the gastric microbiota.

**IMPORTANCE:**

Dysbiosis of the gastric microecology is implicated in various gastric diseases, with *Helicobacter pylori (H. pylori)* infection serving as a pivotal factor influencing the gastric microecological balance and vice versa. We investigated the novel effects of gastric microbiota transplantation (GMT) on gastric microecology and the potential of this treatment to enhance *H. pylori* eradication. GMT significantly enhanced the eradication rate of refractory *H. pylori* infection and improved symptoms in patients with *H. pylori*-positive atrophic gastritis. GMT demonstrated improvements in the cure rate of refractory *H. pylori* infection, potentially offering a new clinical treatment approach. This finding provides new insights and a potential therapeutic direction for treating dysbiosis related chronic gastric diseases.

## INTRODUCTION

Historically, it was believed that the highly acidic environment of the stomach was inhospitable to microorganisms until Marshall and Warren isolated *Helicobacter pylori* (*H. pylori*) from a gastric mucosal tissue sample in 1983 (1). *H. pylori* disrupts the biological barrier and causes bacterial translocation by modulating the local gastric intestinal microecological environment, pH, host immune response, antimicrobial peptides, and cytotoxic factors (2, 3). Furthermore, *H. pylori* infection can lead to varying degrees of dysbiosis in the gastric microbiota (4). Many recent studies have revealed that *H. pylori* eradication leads to the restoration of gastric microbiota diversity and composition (5, 6).

Additionally, the dysbiosis of the gastric microecology induced by *H. pylori* infection represents a potential mechanism for many gastric diseases (4, 7, 8). Presently, strategies for eradicating *H. pylori* primarily rely on combinations of antibiotics and proton pump inhibitors (PPIs) (9, 10); however, these approaches have limitations, including rapid alterations in the gastrointestinal microbiota and a prolonged recovery period to baseline levels (11). Furthermore, because of bacterial resistance to antibiotics, refractory *H. pylori* infections and excessive use of PPIs lead to changes in gastric acidity that continuously disrupt stability within the gastrointestinal microecology (5, 12, 13). Consequently, there is an urgent need to identify safer and more effective antibacterial treatments or regimens, especially for refractory *H. pylori* infections.

Recent studies revealed that the background characteristics of the gastric microbiota are key factors influencing the infection status and rate of eradication of *H. pylori* (14, 15). For example, a high rate of *H. pylori* eradication was associated with the presence of *Rhodococcus*, *Lactobacillus,* and *Sphingomonas* (16). Consequently, “bacteriotherapy” has emerged in recent years as a novel approach for managing *H. pylori* infection by leveraging the inhibitory effects of probiotics (17, 18). Specific probiotic interventions not only enhance the rate of eradication of *H. pylori* but also mitigate the adverse effects associated with antibiotic therapy (19, 20). However, the conventional probiotic formulations typically consist of one or more strains of *lactic acid bacteria*, *Bifidobacterium*, *select streptococci*, and *yeast*, exhibiting a relatively simplistic strain structure with significantly lower diversity than that of the human gastric and intestinal microbiota (21, 22). Consequently, adjunctive probiotic administration does not expedite the restoration of dysbiotic gastric and intestinal microbiota during *H. pylori* treatment (23). Moreover, more solid evidence that probiotic administration enhances the rate of eradication of *H. pylori* is needed (22, 24).

Fecal microbiota transplantation (FMT) has been proven to be a successful approach for altering the microbial composition of a recipient’s intestinal tract and treating various dysbiosis-related diseases (25–28). Interestingly, a recent study reported that the primary pathological features of precancerous lesions can be reproduced by transplanting the gastric microbiota from patients into germ-free mice (29). On the other hand, the administration of gastric commensal microbiota was found to protect the stomach by eliminating IgA-coated bacteria, including pathogenic *H. pylori* (30). Given that the stomach harbors a substantial microbial population and that patients with *H. pylori* infection experience perturbations in their gastric microbiota before and after conventional eradication therapies (3, 15, 18), we introduced the concept of gastric microbiota transplantation (GMT) along with standardized protocols for its implementation. GMT is an innovative therapeutic modality that involves the transfer of donor-derived stomach microbiota into a recipient’s stomach to rectify imbalances within the gastric microflora. To our knowledge, the feasibility, validity, and effectiveness of GMT in patients with refractory *H. pylori* infection and chronic atrophic gastritis have not been reported in the literature.

Therefore, in this study, we assessed the colonization status of GMT in both *H. pylori*-positive and *H. pylori*-negative patients with chronic atrophic gastritis. We further assessed the synergistic therapeutic effects of GMT in patients with refractory *H. pylori* and the influence of this treatment on clinical symptoms and pathological changes in individuals with atrophic gastritis, thereby establishing a foundation for the exploration of novel therapies aimed at modulating gastric microecological balance.

## MATERIALS AND METHODS

### Establishing criteria for the inclusion of participants in the study cohort

#### Inclusion criteria

(i) Participants aged 18–75 years, of any sex; (ii) outpatient or inpatient population requiring endoscopic examination; (iii) no use of antibiotics, antacids, probiotics, or other medications for at least 3 months; (iv) no colonoscopy or similar procedures involving bowel cleansing within the past 2 weeks; and (v) absence of infectious diseases.

#### Exclusion criteria

(i) Individuals under 18 years old or over 75 years old; (ii) individuals with active upper gastrointestinal hemorrhage, pyloric obstruction, or esophageal stenosis; (iii) individuals who had undergone surgery on the esophagus or stomach; (iv) individuals with severe illnesses preventing them from undergoing endoscopy or cooperating with the examination process; (v) pregnant women and lactating mothers; and (vi) individuals who were allergic to the drugs used in this study.

### Study subject selection and grouping

This study was conducted from December 2022 to January 2024 at the First Affiliated Hospital of Chengdu Medical College, where eligible subjects were rigorously selected on the basis of predefined inclusion and exclusion criteria. The clinical data of the subjects were meticulously reviewed and verified, and each participant received comprehensive information regarding the study’s objectives, procedures, potential benefits, and associated risks before providing informed consent. Participants underwent 14C-urea breath testing (14C-UBT) and painless gastroscopy. In the first phase of the study, samples of gastric fluid (GF), gastric mucus layer (GML), and gastric mucosa (GM) (seven cases in each group) were collected from each individual, respectively, who met specific diagnostic criteria for no apparent gastric mucosal lesions as per 14C-UBT diagnostic guidelines (disintegrations per minute [DPM]≤ 99) and the Kyoto Gastritis Classification endoscopic scoring criteria (≤1) (31). The second phase of this investigation involved eight patients with negative *H. pylori* status and 13 patients with positive *H. pylori* status. The third part of this study included seven healthy individuals with negative *H. pylori* status as donors. The recipients were eight patients with atrophic gastritis, of whom five had refractory *H. pylori* infection (defined as two or more failures following standard therapy (32) and three were *H. pylori*-negative. Donor GML was transplanted into the patients, followed by monitoring of clinical symptoms, endoscopic scoring, pathological changes, and *H. pylori* infection status. This research was approved by the Medical Ethics Committee of the First Affiliated Hospital of Chengdu Medical College (no. 2022CYFYIRB-BA-Oct14-01) and registered in the Chinese Clinical Trial Registry (ChiCTR2200066339).

### Collection of the GF, GML, and GM samples

**Fig 1:**
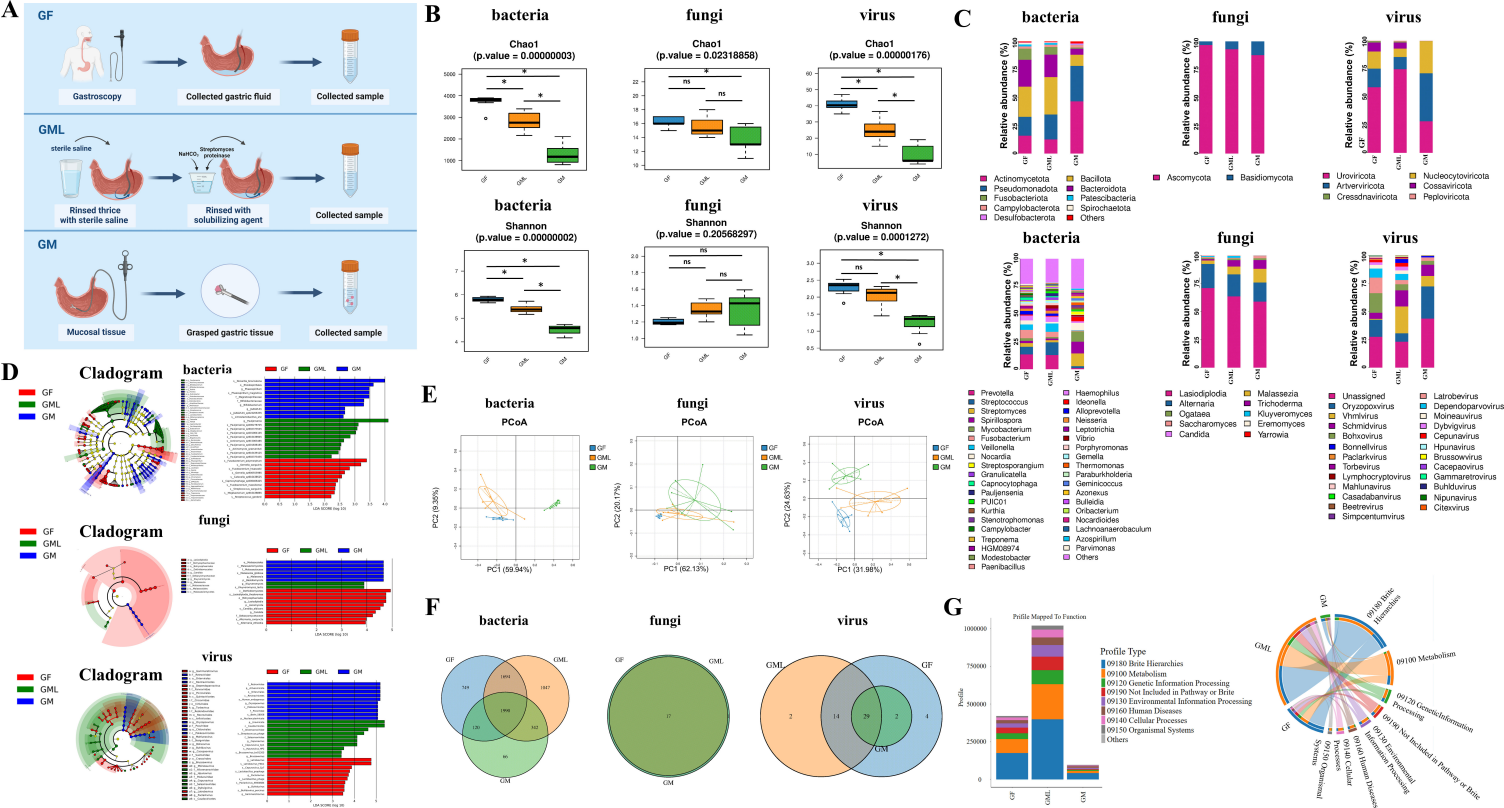
Composition of the gastric microbiota in different parts of the healthy stomach. (A) Schematic representation of the sample collection. Specimens in sequential order: GF, GML, and GM (*n* = 7, respectively). (B) Chao1 and Shannon diversity of the microbiota in GF, GML, and GM: GML exhibited a relatively balanced richness and evenness. (C) Composition of the microbiota in GF, GML, and GM. The upper row showed the distribution at the phylum level, and the lower row showed the genus level. (D) Linear discriminant analysis effect size (LEfSe) showed significant differences in the microbiota in GF, GML, and GM (*P* < 0.05). (E) Principal coordinate analysis (PCoA) showed differences in the composition of bacteria, fungi, and viruses among the three groups. (F) Operational taxonomic unit (OTU) analysis revealed that GML possessed the highest number (1,047) of unique bacterial genera. (G) Functional annotation and differential analysis of the bacteria revealed that the GML exhibited a higher species abundance associated with metabolism, genetic information processing, and cellular processes. *P < 0.05.

As shown in Fig. 1A, following a 6–8 h fast, the following procedures were conducted for each participant: After a 6–8 h fast, samples were collected as follows (Fig. 1A). GF: 50 mL was aspirated via endoscope biopsy channel into a sterile tube. GML: the stomach was rinsed with sterile saline (3×), followed by solubilization (5 min, 40°C) using streptomyces proteinase (20,000 U) and sodium bicarbonate (1 g). The solubilized mucus (50 mL) was collected. GM: after saline rinsing, three antral mucosal biopsies were obtained. All samples were centrifuged (4°C, 3,000 rpm, 10 min), pellets resuspended in saline, flash-frozen in liquid nitrogen, and stored at −80°C.

### Gut microbiota transplantation

As shown in Fig. 3A, the subjects and donors underwent a 6–8 h fast restriction prior to the following procedures: (i) guided by gastroscopy, GF was removed from the donor’s stomach. (ii) The stomach was rinsed with sterile saline three times. (iii) The GML of the donor was thoroughly rinsed with a solubilizing agent and was collected and stored at room temperature. (iv) Subsequently, the GF was suctioned from the recipient’s stomach under gastroscopy guidance, followed by three rinses of the stomach with sterile saline. (v) The GML of the recipient’s stomach was thoroughly dislodged by the washout agent and then completely aspirated. (vi) Next, the GML from the donor was evenly distributed across the entire stomach of the recipient. (vii) The recipient then assumed a right-side lying position and remained prone for more than 1 h. (viii) Images of the stomach, along with samples from both the mucus layer and the gastric mucosa, were collected and preserved.

### Clinical assessment scales

The endoscopic diagnostic criteria were established on the basis of the endoscopic scoring system outlined in the Kyoto Classification of Gastritis (31). These criteria were objectively recorded by the most experienced endoscopist at the Digestive Endoscope Center of the First Affiliated Hospital of Chengdu Medical College. The symptomatological score was derived from the statistical outcomes of the Leeds Dyspepsia Questionnaire and was assessed at 2, 4, and 12 weeks following GMT.

### Metagenomic sequencing

The experimental procedures for sequencing included the following steps: extraction and assessment of metagenomic DNA quality; isolation, purification, and fragmentation of DNA; repair at the ends and addition of “A” at the 3′-end; attachment of sequencing adapters; PCR amplification to produce sequencing libraries; evaluation of library quality; and sequencing on the Illumina HiSeq platform. To improve the accuracy of subsequent bioinformatics analysis and obtain high-quality sequencing data, it was crucial to conduct quality control and screening evaluations of the raw data produced by the sequencing platform. To investigate the microbial composition within the sample, MetaPhlAn 2 software was used to align sequences with evolutionary marker genes specific to reference species, allowing calculation of the evolutionary classification and relative abundance at each taxonomic level for each species.

### Multivariate association

The analytical methods of the Kyoto Encyclopedia of Genes and Genomes (KEGG) function annotation and comparison of species, and functional diversity analysis were detailed in our previous publications (33).

### Statistics and reproducibility

In accordance with the statistical analysis conducted in our prior studies (32), α-Diversity indices, including observed operational taxonomic units (OTUs), the Chao1 index, the Shannon index, and the Simpson index, were computed via QIIME (version 1.9.1). To quantify differences between samples—referred to as β-diversity—principal coordinate analysis (PCoA) and nonmetric multidimensional scaling from the ade4 R package were employed for visualization (33). The classification of the microbiota was conducted following the methodology outlined by Arumugam et al. (34). Briefly, all samples were analyzed via a clustering approach based on metabolite abundance, with partitioning around medoid centroids serving as reference points. The optimal number of clusters was determined via the Calinski-Harabasz index, where higher values indicate superior clustering performance. To assess significant associations between clinical variables—either categorical or continuous—and metabolites, a multivariate association analysis was performed employing MaAsLin as described by Nickols et al. (2024) (35). Spearman’s correlation coefficients were calculated to evaluate the relationships among continuous variables and microbiota composition. Differences in α-diversity indices, genera distributions, and other variables across groups were assessed by using either the Wilcoxon rank-sum test or the Kruskal-Wallis test; *P* values were adjusted according to the Benjamini‒Hochberg procedure for multiple comparisons correction, with significance defined as an adjusted *P* value <0.05.

## RESULTS

### Microbial communities of the GML may play an essential role in the functional ecology of the gastric microbiota

To investigate the composition of the gastric microbiota, we collected samples of GF, GML, and GM from seven healthy individuals for metagenomic analysis (Fig. 1A). α-Diversity analysis revealed that GML presented a relatively balanced richness and evenness (Fig. 1B). At the bacterial phylum level, the dominant phyla across GF, GML, and GM, specifically *Bacillota*, *Bacteroidota*, *Pseudomonadota*, *Actinomycetota*, and *Fusobacteriota*, were consistent. In terms of fungal phyla, all three groups comprised primarily *Ascomycota* and *Basidiomycota*. Among the viral phyla, *Uroviricota*, *Artverviricota*, and *Nucleocytoviricota* predominated in all the groups. At the bacterial genus level, *Prevotella*, *Streptococcus*, *Veillonella*, *Haemophilus*, and *Fusobacterium* were identified as the dominant genera in both the GF and GML groups (Fig. 1C). Linear discriminant analysis effect size (LEfSe) further confirmed significant differences in the microbial abundance distribution among the GF, GML, and GM groups. These findings confirmed that distinct environments harbored unique microbial communities (Fig. 1D).

PCoA further revealed significant differences in the composition of bacteria, fungi, and viruses among the GF, GML, and GM groups (Fig. 1E). OTU analysis revealed that these groups collectively contained 1,990 shared OTUs at the bacterial level. Notably, the GML harbored 1,047 unique OTUs, suggesting that it possessed the greatest number of unique bacterial genera. The rich diversity within these unique genera implied that the GML was characterized by distinct microbial species (Fig. 1F). Additionally, KEGG analysis revealed that the GML group presented greater species abundance associated with metabolism, genetic information processing, and cellular processes (Fig. 1G). Collectively, these findings indicated that the GML plays a pivotal role in the functional ecology of the gastric microbiota and may be an essential site involved in the maintenance of gastric health.

### The GML microbiota is resistant to *H. pylori* infection

**Fig 2:**
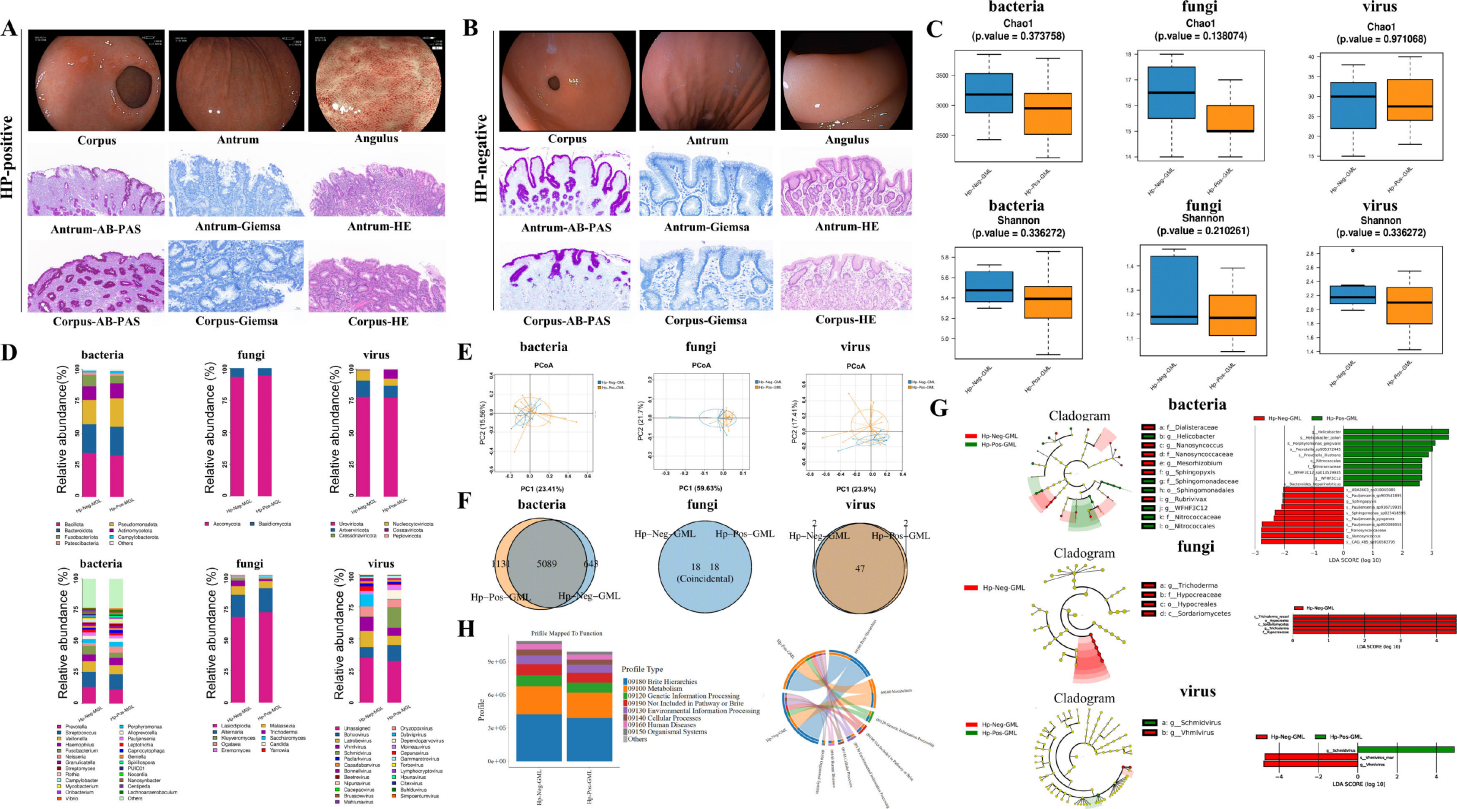
Impact of *H. pylori* infection on the GML microbiome. (A) Endoscopic and pathological examinations of a representative *H. pylori*-positive patient. (B) Endoscopic and pathological examinations of a representative *H. pylori*-negative patient. Panels A and B show that the *H. pylori*-positive patients exhibited more pronounced inflammatory responses. (C) Chao1 and Shannon diversity of the GML microbiota (bacteria, fungi, and viruses) in *H. pylori*-positive and *H. pylori*-negative patients showed no statistically significant differences (*P* > 0.05). (D) The composition of the GML microbiota was similar at the phylum level (upper row) and the genus level (lower row) between *H. pylori*-positive and *H. pylori*-negative patients. (E) PCoA analysis indicated no significant differences between bacterial, fungal, and viral compositions across the *H. pylori*-positive and *H. pylori*-negative groups. (F–G) OTU and LEfSe analysis indicated that the *H. pylori* infection did not significantly alter the structure of the GML microbiome. (H) Functional annotation and differential analysis of bacteria revealed no significant differences between *H. pylori*-positive and *H. pylori*-negative patients based on the KEGG database. Giemsa scale = 50 µm; PAS and HE scales = 20 µm. GML, gastric mucus layer. Hp, *H. pylori*.

To assess the impact of *H. pylori* infection on the GML microbiota, we recruited 13 patients with confirmed *H. pylori* positivity and eight patients who were *H. pylori* negative. Clinical evaluations revealed that *H. pylori*-positive patients exhibited more pronounced inflammatory responses in both endoscopic and pathological examinations (Fig. 2A and B). α-Diversity analysis indicated that microbial richness and diversity in the *H. pylori*-positive group were lower than those observed in the *H. pylori*-negative group; however, this difference was not statistically significant (*P* > 0.05) (Fig. 2C). Species composition analysis revealed that the dominant phyla in both groups included *Bacillota*, *Bacteroidota*, *Pseudomonadota*, *Actinomycetota,* and *Fusobacteriota*, which is consistent with the five major dominant phyla identified in the human stomach (Fig. 2D). Notably, *H. pylori* and *Prevotella illustrans* were significantly enriched within the *H. pylori*-positive group (*P* < 0.05) (Fig. S1). While differences in the average relative abundances of fungi and viruses at the phylum level were noted, these differences were not significant (*P* > 0.05) (Fig. 2D). Collectively, these findings suggested that *H. pylori* infection primarily influenced the relative abundance of gastric microbiota rather than their fundamental composition.

PCoA further revealed no significant differences in bacterial, fungal, or viral composition across the *H. pylori*-positive and *H. pylori*-negative groups (*P* > 0.05) (Fig. 2E). OTU and LEfSe analyses further revealed that although *H. pylori* infection may lead to a decline in the presence or abundance of certain specific species, these findings suggest that *H. pylori* infection does not significantly alter the structure of the gastric microbiota (Fig. 2F and G). At the bacterial level, KEGG database functional annotation and intergroup difference analysis revealed no significant differences between the *H. pylori*-positive and *H. pylori*-negative groups (*P* > 0.05) (Fig. 2H). In summary, while *H. pylori* infection moderately modified the composition of microorganisms in the stomach, but it did not substantially impact the overall function of these microbial populations. This finding may also imply that the gastric microbiota possesses some degree of resistance to *H. pylori* infection. Therefore, whether we can enhance recovery from *H. pylori* infection by reconstructing the microecology of the stomach is a question for consideration.

### GMT improved the clinical symptoms in patients with chronic atrophic gastritis and was synergized to eradicate refractory *H. pylori* infections

**Fig 3:**
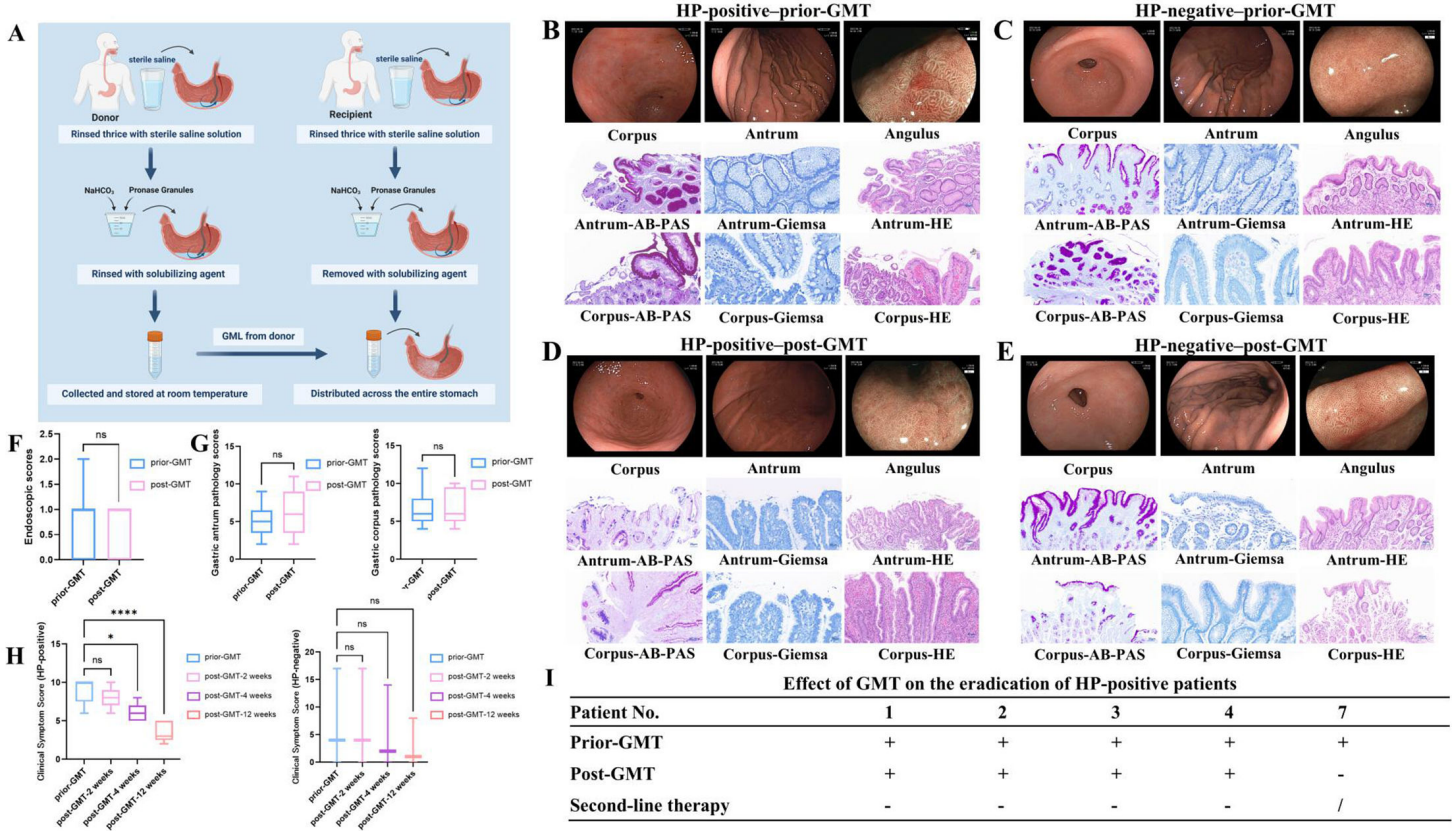
GMT improved clinical symptoms and eradication rates in refractory *H. pylori* infection patients. (A) Schematic diagram of the GMT procedure: collection of the donor’s mucus layer and its transplantation into the recipient’s stomach. (B) Endoscopic and pathological examinations of a representative *H. pylori*-positive patient prior to GMT. (C) Endoscopic and pathological examinations of a representative *H. pylori*-negative patient prior to GMT. (D) Endoscopic and pathological findings of the *H. pylori*-positive patient post-GMT in Fig. 3B. (E) Endoscopic and pathological findings of the *H. pylori*-negative patient post-GMT in Fig. 3C. (F) Endoscopic examinations revealed no significant differences at 4 weeks post-GMT. (G) Pathological examinations of the gastric antrum and corpus revealed no significant differences at 4 weeks post-GMT. (H) Treatment via GMT led to a significant improvement in clinical symptoms in the *H. pylori*-positive patients with chronic atrophic gastritis at 4 weeks (*P* < 0.05) and 12 weeks (*P* < 0.0001). (I) GMT synergized with second-line regimens to effectively eradicate refractory *H. pylori* infections. *P < 0.05, ****P < 0.0001; Giemsa scale = 50 µm; PAS and HE scales = 20 µm. Hp, *H. pylori*.

To evaluate the efficacy of GMT in synergizing treatment to eradicate refractory *H. pylori* infection and improving clinical symptoms in patients with chronic atrophic gastritis, we enrolled eight participants, comprising five individuals with refractory *H. pylori* infections and three *H*. *pylori*-negative individuals. We performed the initial clinical application of GMT by transplanting normal human GML into the stomachs of these patients via endoscopy (Fig. 3A). Our findings revealed no significant improvement in either the Kyoto gastritis score or the pathological score among either *H. pylori*-positive or *H. pylori*-negative patients at 4 weeks after the GMT procedure (Fig. 3B through G). Nevertheless, there was a notable improvement in clinical symptoms following GMT, particularly among *H. pylori*-positive patients who exhibited more pronounced symptom relief at 12 weeks (*P* < 0.0001) (Fig. 3H).

One *H*. *pylori*-positive patient tested negative on a 14C-UBT test after GMT; however, the remaining four patients continued to test positive and subsequently underwent second-line quadruple therapy for refractory *H. pylori* infection (esomeprazole 20 mg bid + colloidal bismuth subcitrate 250 mg bid + amoxicillin 1 g bid + levofloxacin 0.5 g qd), which resulted in all patients achieving negative 14C-UBT tests one month later (Fig. 3I). Therefore, GMT represented a novel therapeutic approach capable of enhancing clinical outcomes for chronic atrophic gastritis patients while synergizing with second-line regimens to eradicate refractory *H. pylori* infections effectively.

### GMT facilitated the restoration of the gastric microbiota in *H. pylori*-positive patients

**Fig 4:**
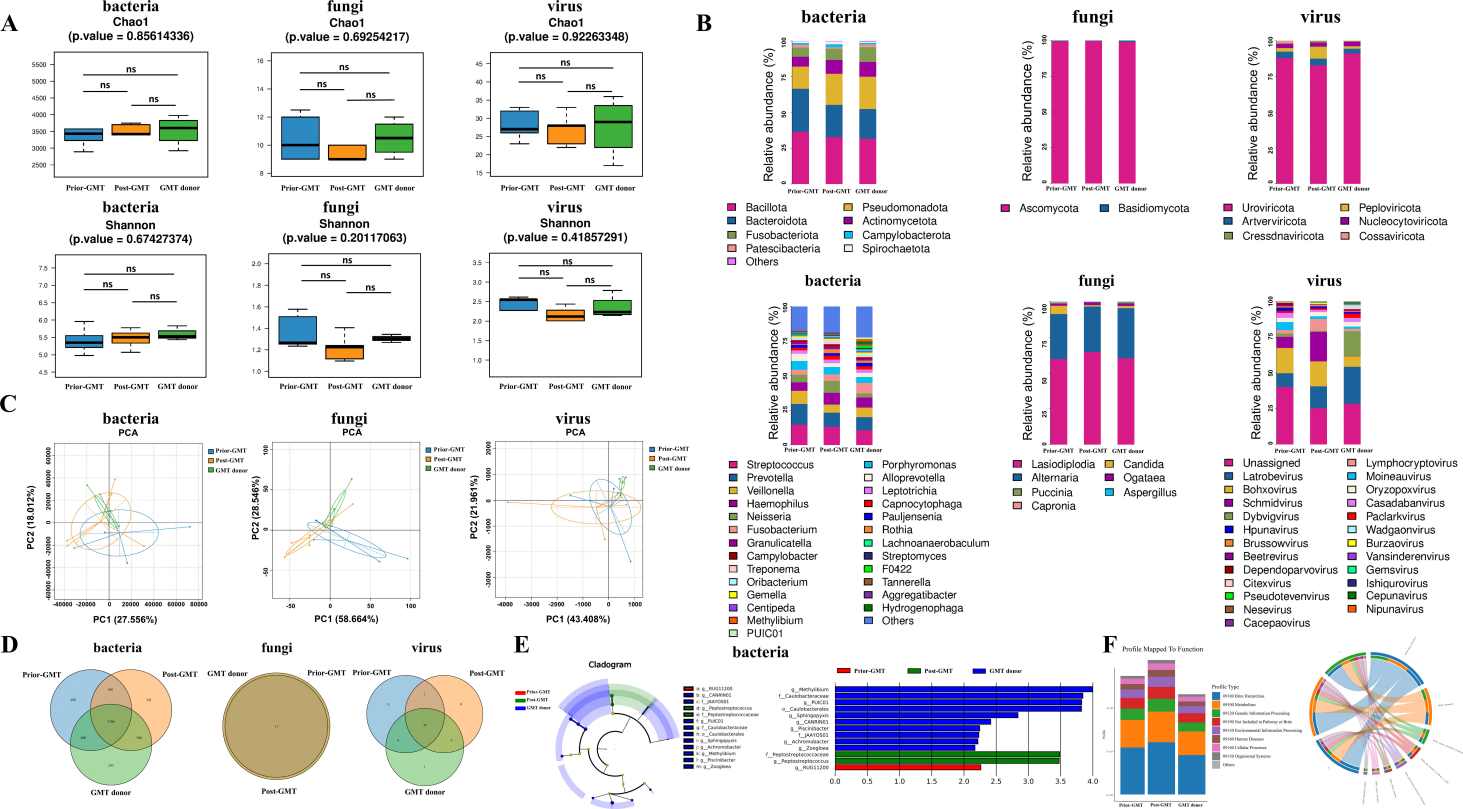
Impact of GMT on the composition of the gastric microbiome in *H. pylori*-positive patients. (A) Chao1 and Shannon diversity of the gastric microbiota in *H. pylori*-positive patients showed that the bacterial species richness increased following GMT, although this difference was not statistically significant (*P* > 0.05). (B) At 4 weeks post-GMT, the bacterial species abundance in *H. pylori*-positive patients at both the phylum level (upper row) and the genus level (lower row) tended to align more closely with the donor group. (C) PCA analysis revealed that the bacteria in *H. pylori*-positive patients post-GMT were similar to those in the respective donors. (D–E) OTU and LEfSe analysis showed that the recipients acquired a significant number of unique bacterial strains from their respective donors. (F) Functional annotation and differential analysis of the bacterial taxa indicated that the microbial compositions related to Brite hierarchies and metabolic functions in the *H. pylori*-positive patients post-GMT increasingly resembled those of the respective donors.

To further evaluate the specific impact of GMT on the gastric microbiota, we conducted a metagenomic analysis among normal donors, *H. pylori*-positive patients prior to GMT, and 1 month post-GMT (*n* = 5, respectively). α-Diversity analysis revealed that bacterial species richness in the *H. pylori*-positive patient group increased following GMT, approaching the levels observed in donors (Table S1); however, this difference was not statistically significant (*P* > 0.05) (Fig. 4A). We noted that the dominant phyla of the microbiota prior to and post-treatment with GMT remained *Bacillota*, *Bacteroidota*, *Pseudomonadota*, *Actinomycetota*, and *Fusobacteriota*, although differences in species abundance were evident. After GMT, the bacterial species abundances at both the phylum and genus levels in *H. pylori*-positive patients tended to align more closely with those in the donor group (Fig. 4B). Principal component analysis (PCA) indicated that the bacteria present in *H. pylori*-positive patients post-GMT were similar to those found in donors (Fig. 4C). These findings suggest that microbiota transplantation can effectively alter the structure of the recipient’s gastric microbiota toward characteristics typical of healthy donors.

OTU and LEfSe analyses further demonstrated substantial differences in microbial composition between *H. pylori*-positive patients prior to GMT treatment and donors; however, post-GMT, these patients acquired a significant number of unique bacterial strains from their respective donors (Fig. 4D and E). Additionally, we conducted a MetaStat analysis that provided additional evidence that recipients of GMT have assimilated distinct microbial populations from the donors (Fig. S2). We performed functional annotation and intergroup difference analysis at the KEGG database level for the bacterial communities. The results indicated that the microbial compositions related to Brite hierarchies and metabolic functions increasingly resembled those of donors after treatment via GMT (Fig. 4F). These findings suggested that post-GMT microecology for *H. pylori*-positive patients converges toward donor profiles while achieving objectives related to reconstructing gastric microecology.

### GMT influenced the species richness of the gastric microbiota in *H. pylori*-negative patients

**Fig 5:**
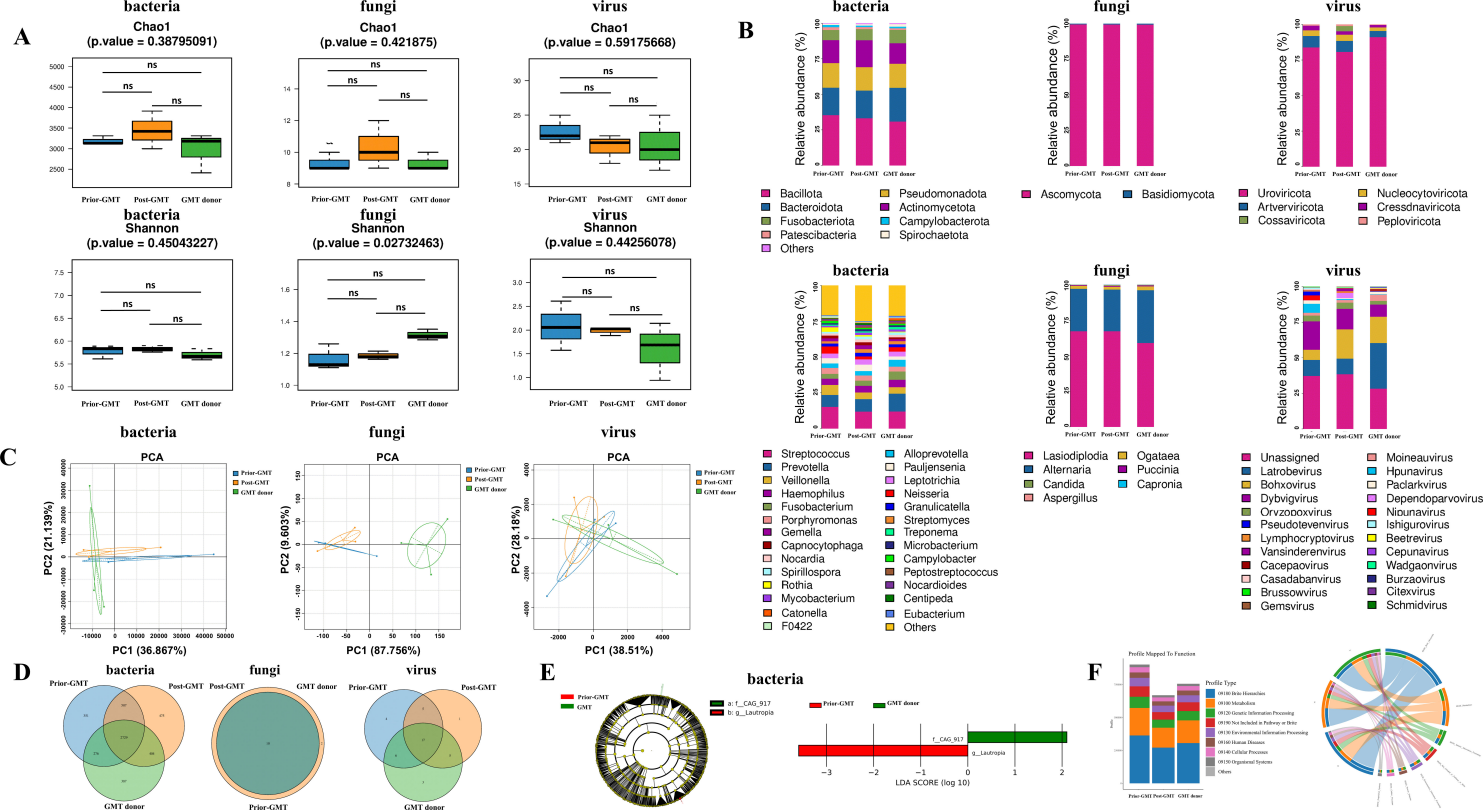
Impact of GMT treatment on the composition of the gastric microbiome in *H. pylori*-negative patients. (A) Chao1 and Shannon diversity of the gastric microbiota in the *H. pylori*-negative patients showed that the species richness of bacteria and fungi increased following GMT treatment, although this difference was not statistically significant (*P* > 0.05). (B) At 4 weeks post-GMT treatment, the bacterial species abundance in *H. pylori*-negative patients at both the phylum level (upper row) and the genus level (lower row) showed no significant changes. (C) PCA analysis revealed that the bacterial and viral compositions of *H. pylori*-negative patients post-GMT were comparable to those of the donors, whereas the changes in fungal composition were minimal. (D–E) OTU and LEfSe analyses indicated that *H. pylori*-negative patients exhibited multiple independent microbial species relative to the donors. (F) Functional annotation and differential analysis of the bacterial taxa by using the KEGG database revealed no significant functional changes post-GMT.

We subsequently analyzed the microbial composition of *H. pylori*-negative recipients prior to and post-GMT (*n* = 3) as well as that of their donors (*n* = 3). α-Diversity analysis revealed an increase in species richness within the *H. pylori*-negative patients following GMT; however, this difference was not statistically significant (*P* > 0.05) (Fig. 5A). Concurrently, species composition analysis revealed no significant changes in gastric microecology at either the phylum or the genus level prior to or post-GMT, although differences in the abundances of specific species were observed (Fig. 5B). PCA demonstrated that the bacterial and viral compositions of *H. pylori*-negative patients post-GMT were comparable to those of the donors, whereas the changes in fungal composition were minimal (Fig. 5C). OTU and LEfSe analyses further revealed that *H. pylori*-negative patients presented multiple independent microbial species relative to those in their donors, with several donor-derived species acquired following GMT (Fig. 5D and E). Additionally, MetaStat analysis provided additional evidence that recipients of GMT assimilated distinct microbial populations from the donors (Fig. S3). Functional annotation and intergroup difference analysis at the bacterial level via the KEGG database revealed no significant functional changes prior to or post-GMT (Fig. 5F), suggesting that GMT primarily influenced species abundance among *H. pylori*-negative patients without significantly altering functional characteristics.

## DISCUSSION

Currently, research on gastric microecology is rare and has focused primarily on the gastric juice and gastric mucosa; moreover, little is known about the impact of *H. pylori* infection on the microbial community of the GML (36–38). Our findings confirmed the substantial presence of microorganisms in the GML, with varying proportions of bacteria, fungi, and viruses in the GF, GML, and GM. Significant differences in species richness and diversity were detected among these three groups. A previous mouse study suggested that most bacteria reside within gastric pits or adhere to epithelial cells beneath a protective mucus layer (39). Our study also revealed significantly lower species richness and bacterial diversity within the GM than in the GML. Therefore, further investigations into both the composition and function of the GML, which serves as a crucial barrier, are highly important.

To date, the effects of *H. pylori* infection on the richness, diversity, and community structure of the gastric microbiota have not been definitively established (6, 8, 40). For the first time, we analyzed mucus layer samples from 13 *H*. *pylori*-positive and eight *H*. *pylori*-negative patients. Analysis at the phylum level revealed that both groups were predominantly composed of *Bacillota, Bacteroidota, Pseudomonadota, Actinomycetota,* and *Fusobacteriota*, which are phyla commonly found in the human stomach. Most studies indicated that both *H. pylori*-positive and *H. pylori*-negative individuals harbor similar phyla but with varying relative abundances (40, 41). However, other studies have shown that *H. pylori* infection significantly reduces species diversity and richness (11, 42). The differences in the conclusions of these studies may be related to individual variability, differences in race, geographic location, diet, sample size, and the use of different research methods. Our study revealed that from the perspective of species differences, the α-diversity analysis suggested that the bacterial, fungal, and viral richness and diversity of the gastric microbiota in *H. pylori*-positive patients were lower than those in the *H. pylori*-negative group; however, this difference was not significant (*P* > 0.05). β-Diversity analysis revealed that there was no significant difference in the composition of the gastric microbiota between the two groups (*P* > 0.05). We subsequently used MetaStat analysis to analyze the differences at the genus and phylum levels between the two groups and found that there were three significantly different genera: *Helicobacter*, *Anaeroglobus*, and *Paraburkholderia*. Among these genera, *Helicobacter* and *Paraburkholderia* were significantly more abundant in the *H. pylori*-positive group than in the *H. pylori*-negative group, whereas *Anaeroglobus* was significantly more abundant in the *H. pylori*-negative group (*P* < 0.05).

The compositional characteristics of fungi and viruses within the healthy human stomach remain unclear because of a lack of well-characterized fungal and viral sequences in reference databases (43–45). Our study identified *Ascomycota* and *Basidiomycota* as prevalent fungal phyla in both *H. pylori*-positive and *H. pylori*-negative groups. Previous research on fungal microbiota in gastric cancer has also shown that *Ascomycota* and *Basidiomycota* are the dominant phyla across healthy individuals as well as between gastric cancer tissues and adjacent tissues (44). Our findings suggested that these two phyla were dominant regardless of the accompanying *H. pylori* infection; this discovery provides insight into the characteristic features of fungal compositions within the stomach. At the viral phylum level, *Uroviricota*, *Artverviricota*, and *Nucleocytoviricota* were identified as the dominant viral phyla in both groups. Currently, there are no studies characterizing the viral composition within the stomach; thus, our results provide a valuable reference for future research.

Notably, we attempted to manage refractory *H. pylori* infection with atrophic gastritis by modulating the gastric microecological environment. In parallel with FMT, GMT was successfully established to enhance the eradication of *H. pylori* infection involving atrophic gastritis. This was the first successful approach for altering the gastric microbial composition and has been applied to treat gastric diseases by GMT. However, the endoscopic and pathological assessment revealed insignificant amelioration, possibly due to an inadequate duration of observation. The precise mechanism by which GMT enhanced the efficacy of *H. pylori* eradication remains unclear. Satoh-Takayama and colleagues demonstrated that the second dominant type of innate lymphoid cell in the stomach was induced by commensal microbes and provides protection against *H. pylori* infection through B-cell activation and IgA production (30). While Satoh-Takayama et al. demonstrated ILC2-mediated protection against *H. pylori* in mice, this mechanism remains hypothetical in human GMT and requires validation through future mechanistic studies or animal models. Furthermore, our study revealed that compared with the microbiome of their *H. pylori*-negative counterparts, the microbiota of *H. pylori*-positive patients with atrophic gastritis was more similar to that of healthy donors after GMT. A potential explanation is that the loss of acid-secreting parietal cells induced by *H. pylori* infection may provide a favorable environment for colonization by the transplanted gastric microbiome. Another possible reason is that the species richness and diversity of the *H. pylori*-positive gastric microbiota were lower than those of the *H. pylori*-negative microbiota (although not significantly different), with the former creating a relatively friendly environment for foreign microorganisms to colonize.

However, research on this topic remains incipient and faces several challenges: (i) microbial complexity: microbial suspensions sourced from gastric mucus were employed for GMT therapy. Nevertheless, introducing donor microbes into the recipients’ hostile acidic milieu while acclimatizing presents formidable challenges. (ii) Technical intricacy: GMT involves invasive measures such as endoscopic biopsies, sample collection involving patient fasting, and sedation/anesthesia. Given the similarity between the microbial communities of the stomach and oral cavity (46), the administration of oral microbiota may be considered as a therapeutic option in our future studies. (iii) Standardization gaps: the GMT procedure remains in its nascent stages, characterized by a lack of uniform operational guidelines and protocols. The best practices regarding sample collection, quality control, optimal dosages, frequency, and routes of administration remain ambiguous. (iv) Safety considerations: the multi-microorganism involvement inherent within GMT raises concerns regarding infectious/allergic/rejection phenomena. Our preliminary investigations in a small sample suggested the procedural safety of GMT, which yielded no major adverse events.

Limitations of this pilot study include the small sample size of GMT patients, short follow-up periods, and absence of a sham-transplantation control group. Additional clinical studies with a larger sample size and adequate duration of observation are essential to prove the effectiveness of GMT, and further foundational research is necessary to clarify the mechanisms underlying the enhancement of *H. pylori* infection eradication through modulation of the gastric microbiota. Nevertheless, our pilot study demonstrated for the first time that GMT was a novel and successful approach that can be applied to modulate the gastric microbial diversity or structures associated with various gastric diseases.

## Data Availability

The datasetsdata sets presented in this study can be found in online repositories. The names of the repository/repositories and accession number(s) can be found below: GSA ID: CRA020254 and submit ID: subCRA032707.

## SUPPLEMENTAL MATERIAL

Figure S1 spectrum.03263-24-s0001.TIF Microbial taxonomic differences between *H. pylori*-positive and H. pylori-negative patients by MetaStat analysis.

Figure S2 spectrum.03263-24-s0002.tif Microbial taxonomic shifts in *H. pylori*-positive patients before and after GMT, compared with donors.

Figure S3 spectrum.03263-24-s0003.TIF Microbial taxonomic shifts in *H. pylori*-negative patients before and after GMT, compared with donors.

Supplemental figure legends spectrum.03263-24-s0004.docx Legends for Fig. S1 to S3.

Table S1 spectrum.03263-24-s0005.docx Characteristic taxa and abundance changes in patients before and after GMT.

ASM does not own the copyrights to Supplemental Material that may be linked to, or accessed through, an article. The authors have granted ASM a non-exclusive, world-wide license to publish the Supplemental Material files. Please contact the corresponding author directly for reuse.
