# Supplementary figures and images for: Gastric microbiota transplantation enhanced the eradication of refractory Helicobacter pylori infection by modulating the gastric microbiota: a pilot study
Source: Microbiol Spectr. 2025 Aug 18;13(10):e03263-24. doi: 10.1128/spectrum.03263-24 (PMC12502787; doi:10.1128/spectrum.03263-24)

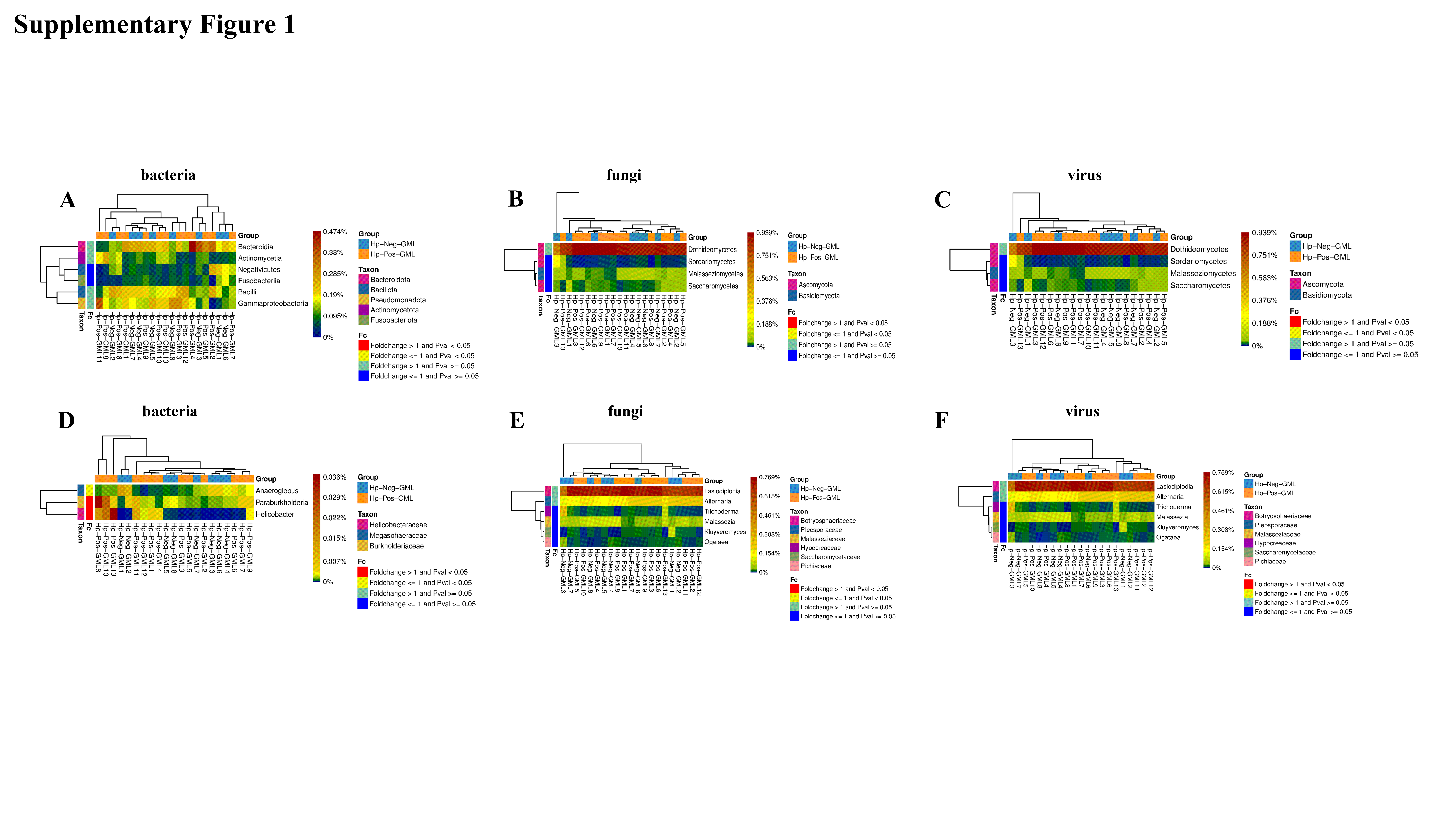

Supplement: Figure S1 — Microbial taxonomic differences between H. pylori-positive and H. pylori-negative patients by MetaStat analysis. [file spectrum.03263-24-s0001.tif]

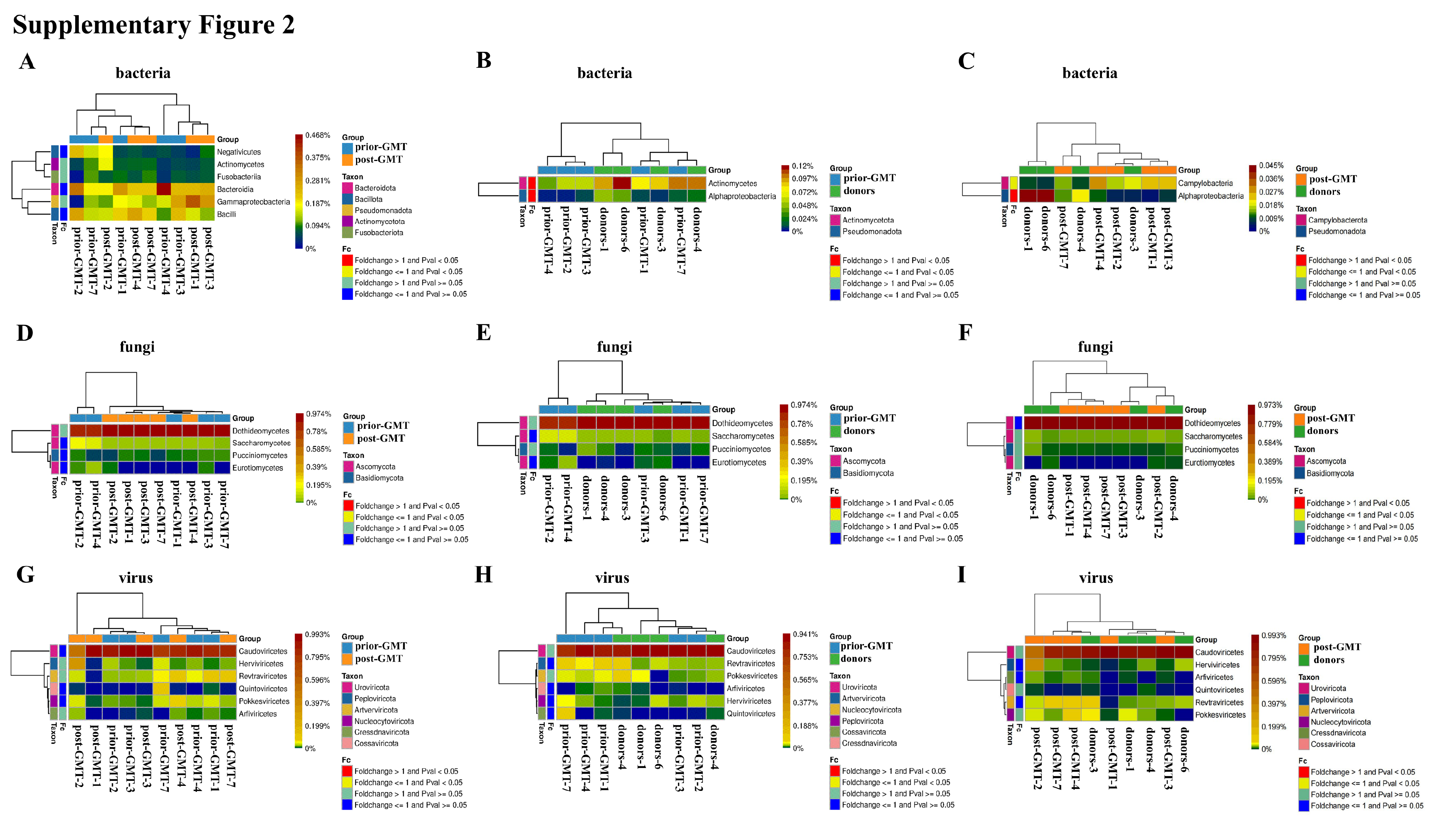

Supplement: Figure S2 — Microbial taxonomic shifts in H. pylori-positive patients before and after GMT, compared with donors. [file spectrum.03263-24-s0002.tif]

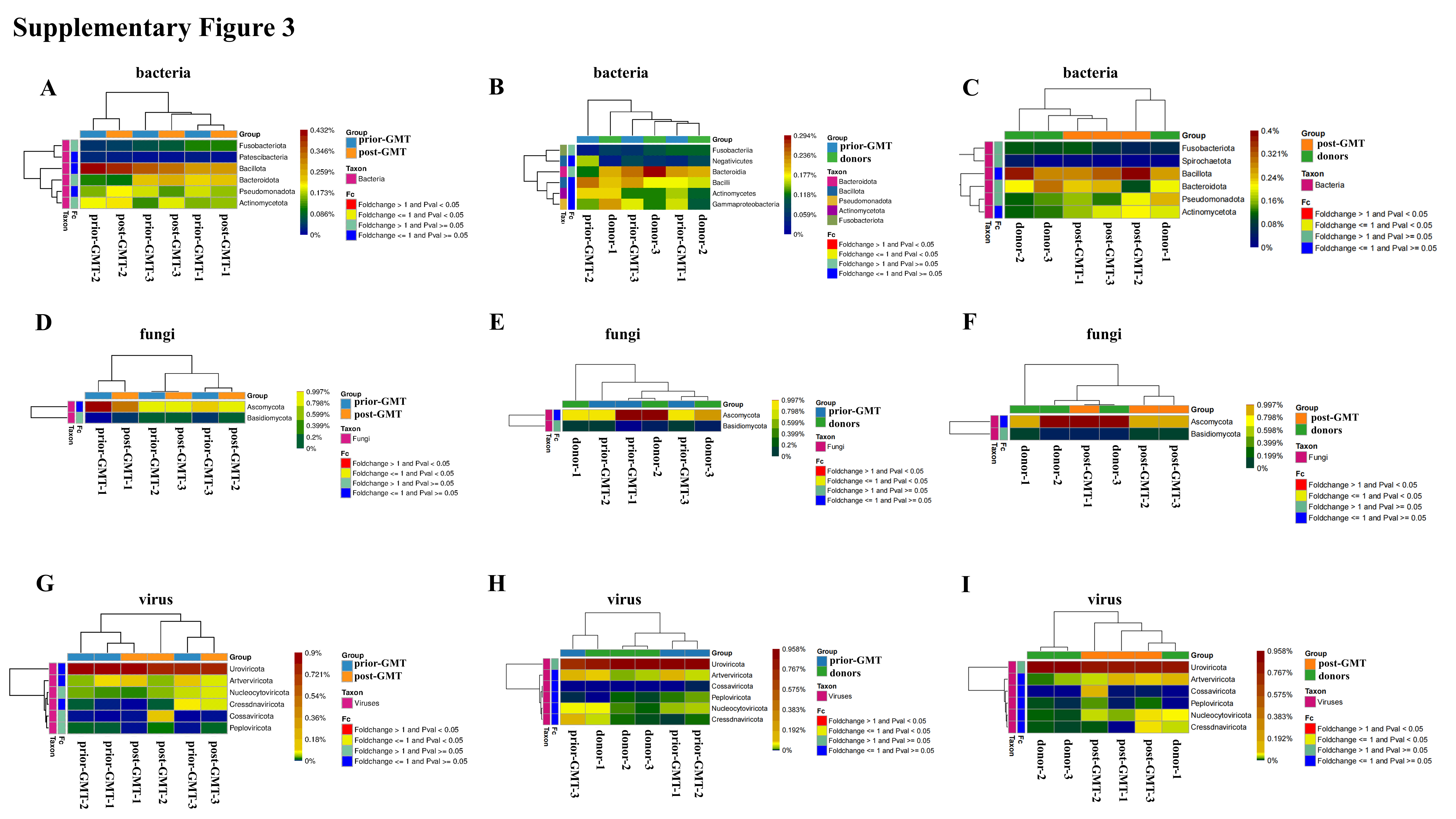

Supplement: Figure S3 — Microbial taxonomic shifts in H. pylori-negative patients before and after GMT, compared with donors. [file spectrum.03263-24-s0003.tif]
