# Supplementary material for: Gastric microbiota transplantation enhanced the eradication of refractory Helicobacter pylori infection by modulating the gastric microbiota: a pilot study
Source: Microbiol Spectr. 2025 Aug 18;13(10):e03263-24. doi: 10.1128/spectrum.03263-24 (PMC12502787; doi:10.1128/spectrum.03263-24)
Supplement: Supplemental figure legends — Legends for Fig. S1 to S3. [file spectrum.03263-24-s0004.docx]

Supplementary figure legends

Supplementary Figure 1. Microbial taxonomic differences between *H. pylori*-positive and *H. pylori*-negative patients by MetaStat analysis. A. Bacterial phylum level showed that the richness of *H. pylori*-positive and *H. pylori*-negative patients were not statistically significant (*P*>0.05). B. Fungal phylum level showed that the richness of *H. pylori*-positive and *H. pylori*-negative patients were not statistically significant (*P*>0.05). C. Viral phylum level showed that the richness of *H. pylori*-positive and *H. pylori*-negative patients were not statistically significant (*P*>0.05). D. Bacterial genus level showed that the richness of *H. pylori*-positive and *H. pylori*-negative patients were statistically significant. *Helicobacter* and *Paraburkholderia* were enriched in *H. pylori*-positive patients, whereas *Anaeroglobus* was more abundant in *H. pylori*-negative patients (*P*＜0.05). E. Fungal genus level showed that the richness of *H. pylori*-positive and *H. pylori*-negative patients were not statistically significant (*P*>0.05). F. Viral genus level showed that the richness of *H. pylori*-positive and *H. pylori*-negative patients were not statistically significant (*P*>0.05).

Supplementary Figure 2. Microbial taxonomic shifts in *H. pylori*-positive patients before and after GMT, compared with donors. A. Bacterial phylum level showed that the richness of prior- and post-GMT and donors were not statistically significant (*P*>0.05). B. Bacterial phylum level showed that the richness of prior-GMT and donors were statistically significant, with lower *Actinomycetes* and *Alphaproteobacteria* in prior-GMT patients (*P*＜0.05). C. Bacterial phylum level showed that the richness of post-GMT and donors were not statistically significant (*P*>0.05). D-F. Fungal phylum level showed that the richness of prior-, post-GMT and donors were not statistically significant (*P*>0.05). G-I. Viral phylum level showed that the richness of prior-, post-GMT and donors were not statistically significant (*P*>0.05).

Supplementary Figure 3. Microbial taxonomic shifts in *H. pylori*-negative patients before and after GMT, compared with donors. A-C. Bacterial genus level showed that the richness of prior-, post-GMT and donors were not statistically significant (*P*>0.05). D-F. Fungal genus level showed that the richness of prior-, post-GMT and donors were not statistically significant (*P*>0.05). G-I. Viral genus level showed that the richness of prior-, post-GMT and donors were not statistically significant (*P*>0.05).
