# Supplementary material for: Gastric microbiota transplantation enhanced the eradication of refractory Helicobacter pylori infection by modulating the gastric microbiota: a pilot study
Source: Microbiol Spectr. 2025 Aug 18;13(10):e03263-24. doi: 10.1128/spectrum.03263-24 (PMC12502787; doi:10.1128/spectrum.03263-24)
Supplement: Table S1 — Characteristic taxa and abundance changes in patients before and after GMT. [file spectrum.03263-24-s0005.docx]

| Supplementary Table 1. Characteristic Taxa and Abundance Changes in Patients Before and After GMT | | | | |
| --- | --- | --- | --- | --- |
| Taxonomic Level | Taxon | Prior-GMT | Post-GMT | GMT donor |
| Phylum | Bacillota | 36.48147773 | 32.75204636 | 31.54763 |
| Phylum | Bacteroidota | 30.18584571 | 22.58906193 | 20.90351 |
| Phylum | Pseudomonadota | 15.42930822 | 21.75264035 | 22.63463 |
| Phylum | Actinomycetota | 6.964838827 | 9.727576965 | 10.24234 |
| Phylum | Fusobacteriota | 6.374791742 | 7.789322709 | 10.33872 |
| Phylum | Patescibacteria | 1.923448026 | 1.05445853 | 1.920753 |
| Phylum | Campylobacterota | 1.332270916 | 2.092575154 | 1.170699 |
| Phylum | Spirochaetota | 1.074683086 | 1.917015574 | 0.696849 |
| Phylum | Others | 0.233335748 | 0.325302427 | 0.544875 |
| Genus | Streptococcus | 14.6550959797175 | 13.0227888446215 | 10.6391886997465 |
| Genus | Prevotella | 15.0171097428468 | 10.3225498007968 | 9.44650488953278 |
| Genus | Veillonella | 9.46671495834842 | 5.90467222021007 | 6.96736689605215 |
| Genus | Haemophilus | 6.17692140528794 | 8.46220934444042 | 7.24357116986599 |
| Genus | Neisseria | 5.49085114089098 | 8.617747193 | 2.91180731618979 |
| Genus | Fusobacterium | 3.59084389713872 | 4.77542919232162 | 7.64893154654111 |
| Genus | Porphyromonas | 6.34494748279609 | 5.24195581311119 | 4.29311843534951 |
| Genus | Alloprevotella | 5.2580659181456 | 2.76192683810214 | 2.96352770735241 |
| Genus | Leptotrichia | 2.3419340818544 | 2.59532053603767 | 2.41593625498008 |
| Genus | Capnocytophaga | 1.95013400941688 | 2.84780876494024 | 2.24961970300616 |
| Genus | Pauljensenia | 2.09819630568635 | 1.82102136906918 | 2.57942774357117 |
| Genus | Rothia | 0.523201738500543 | 2.93783411807316 | 1.64320898225281 |
| Genus | Granulicatella | 1.6491126403477 | 2.08283955088736 | 1.2662803332126 |
| Genus | Campylobacter | 1.18470119521912 | 1.42455632017385 | 1.12962694675842 |
| Genus | Treponema | 1.05932633103948 | 1.90044186888808 | 0.682832307135096 |
| Genus | Oribacterium | 1.02105034407823 | 0.881303875407461 | 1.48304961970301 |
| Genus | Gemella | 0.786961245925389 | 1.14167330677291 | 1.20456356392611 |
| Genus | Centipeda | 0.561680550525172 | 1.22210793190873 | 1.10050706265846 |
| Genus | Lachnoanaerobaculum | 0.737269105396596 | 0.905526982977182 | 1.23462513582035 |
| Genus | Streptomyces | 0.645679101774719 | 0.771836291198841 | 1.10318725099602 |
| Genus | F0422 | 0.628033321260413 | 0.242520825787758 | 1.51528431727635 |
| Genus | Tannerella | 0.672944585295183 | 0.481883375588555 | 1.02136906917783 |
| Genus | Aggregatibacter | 0.687953639985513 | 1.00575153929736 | 0.360557768924303 |
| Genus | Hydrogenophaga | 0.106975733429917 | 0.111930459978269 | 1.70340456356393 |
| Genus | Methylibium | 0.108656283955089 | 0.0688735965229989 | 1.73665338645418 |
| Genus | PUIC01 | 0.0517783411807316 | 0.0658601955813111 | 1.23687069902209 |
| Genus | Others | 17.1838609199565 | 18.3816298442593 | 22.2189786309308 |
